# GLP-1R signaling modulates colonic energy metabolism, goblet cell number and survival in the absence of gut microbiota

**DOI:** 10.1016/j.molmet.2024.101924

**Published:** 2024-03-21

**Authors:** Thomas U. Greiner, Ara Koh, Eduard Peris, Mattias Bergentall, Malin E.V. Johansson, Gunnar C. Hansson, Daniel J. Drucker, Fredrik Bäckhed

**Affiliations:** 1 Wallenberg Laboratory, Department of Molecular and Clinical Medicine, Institute of Medicine, University of Gothenburg, Sweden; 2 Department of Life Sciences, Pohang University of Science and Technology, Pohang 37673 South Korea; 3 Department of Medical Biochemistry and Cell Biology, Institute of Biomedicine, University of Gothenburg, Sweden; 4 Department of Medicine, Lunenfeld-Tanenbaum Research Institute, Mount Sinai Hospital, University of Toronto, Toronto ON, Canada; 5 Novo Nordisk Foundation Center for Basic Metabolic Research, Faculty of Health Sciences, University of Copenhagen, Denmark; 6 Region Västra Götaland, Sahlgrenska University Hospital, Department of Clinical Physiology, Gothenburg, Sweden

**Keywords:** GLP-1, Goblet cell, Microbiota, ER-stress, Mitochondria, Respiration

## Abstract

**Objectives:**

Gut microbiota increases energy availability through fermentation of dietary fibers to short-chain fatty acids in conventionally raised mice. Energy deficiency in germ-free (GF) mice increases glucagon-like peptide-1 (GLP-1) levels, which slows intestinal transit. To further analyze the role of GLP-1-mediated signaling in this model of energy deficiency, we re-derived mice lacking GLP-1 receptor (GLP-1R KO) as GF.

**Methods:**

GLP-1R KO mice were rederived as GF through hysterectomy and monitored for 30 weeks. Mice were subjected to rescue experiments either through feeding an energy-rich diet or colonization with a normal cecal microbiota. Histology and intestinal function were assessed at different ages. Intestinal organoids were assessed to investigate stemness.

**Results:**

Unexpectedly, 25% of GF GLP-1R KO mice died before 20 weeks of age, associated with enlarged ceca, increased cecal water content, increased colonic expression of apical ion transporters, reduced number of goblet cells and loss of colonic epithelial integrity. Colonocytes from GLP-1R KO mice were energy-deprived and exhibited increased ER-stress; mitochondrial fragmentation, increased oxygen levels and loss of stemness. Restoring colonic energy levels either by feeding a Western-style diet or colonization with a normal gut microbiota normalized gut phenotypes and prevented lethality.

**Conclusions:**

Our findings reveal a heretofore unrecognized role for GLP-1R signaling in the maintenance of colonic physiology and survival during energy deprivation.

## Highlights

• Deletion of GLP-1R in germ-free mice is associated with increased lethality.
• Germ-free GLP-1R KO mice have a reduced number of goblet cells in the colon.
• GLP-1R signaling is essential for maintaining mitochondrial function during energy deprived states.
• Absence of GLP-1R signaling in germ-free mice reduces stemness in colonic crypts.

## 1 Introduction

Glucagon-like peptide 1 (GLP-1) is an important gut derived hormone that enables nutrient assimilation. There are several well-established actions of GLP-1, including stimulation of postprandial insulin secretion from β cells supporting development of GLP-1 medicines for type 2 diabetes (T2D), and reduction of appetite, enabling weight loss and approval of GLP-1-based medicines for the treatment of people with obesity [1]. The expanding actions and improved efficacy of modern GLP-1 receptor agonists (GLP-1RAs) have led to increasing utilization of these medicines for people with T2D and obesity [[2], [3], [4], [5], [6]]. Moreover, the safety of these medicines has been reinforced by data from cardiovascular outcome trials demonstrating reductions in rates of non-fatal myocardial infarction, non-fatal stroke, cardiovascular death and all-cause mortality [7,8].

Beyond the pharmacological actions of GLP-1 on islet cells and appetite centres in the brain, GLP-1 also exerts direct and indirect effects on the gastrointestinal tract, including reduction of gastric emptying and small bowel motility. These actions are predominantly mediated by neural pathways [9], which together with the central aversive actions of GLP-1RAs, contribute to the common side effects of these medicines, including nausea, vomiting, diarrhea, and constipation [10].

In the gut, GLP-1 acts pharmacologically as a small bowel growth factor, via promotion of crypt fission [11], and also controls local intestinal immune function, via GLP-1 receptors (GLP-1R)s on subsets of small bowel intraepithelial lymphocytes (IELs) [12]. Intriguingly, genetic elimination of the IEL GLP-1R is associated with altered gut microbiota in mice treated with GLP-1RAs [13], highlighting potential pathways linking systemic GLP-1 action to modulate gut microbiota and local intestinal inflammation.

Gut microbiota has emerged as a regulator of many different aspects of host functions including the maturation of the immune system [14] and host metabolism [15]. Gut microbiota also has local effects in the intestine where it affects intestinal architecture by modulating vascular remodeling [16,17] as well as proliferation and differentiation of intestinal stem and progenitor cells [[18], [19], [20]]. The microbiota contributes to increased energy uptake by extracting energy from otherwise indigestible polysaccharides in the colon through the fermentation into short chain fatty acids (SCFAs) [21]. Metabolism of the SCFA butyrate, the preferred energy source for colonocytes, generates a hypoxic colonic environment by reducing oxygen levels following mitochondrial β-oxidation, which further favors the oxygen-intolerant butyrate producing bacteria [22]. In addition, SCFAs serve as signaling molecules, by binding to G protein-coupled receptors (GPCRs), that regulate GLP-1 secretion from L-cells [23,24].

We have previously shown that physiological levels of endogenous GLP-1 are increased in germ-free (GF) mice, which are energy-deprived due to low levels of SCFAs. Indeed, multiple strains of microbiota have been postulated to enhance gut GLP-1 secretion through local interactions, actions mediated through microbial metabolites or peptides [[23], [24], [25], [26], [27], [28]]. Elevated GLP-1 levels in turn may tune epithelial integrity and immunity, while serving as a break, slowing down intestinal transit to allow increased energy harvest [29]. Conversely, microbial metabolites have also been shown to inhibit GLP-1 secretion [30,31]. Hence the extent of positive and negative microbial-derived regulators likely contributes to L-cell secretory tone, and the circulating levels of GLP-1. We previously demonstrated that mice lacking GLP-1 receptor (GLP-1R KO) exhibit marked shifts in the proportions of gut microbiota, in association with dysregulated immune-related gene expression in the gut, and enhanced sensitivity to intestinal injury [12]. To provide increased understanding of the importance of gut microbiota in the presence or absence of GLP-1R signaling we re-derived and assessed key phenotypes of GLP-1R KO GF mice, with a focus on the gut epithelium.

## 2 Materials and methods

### 2.1 Mice

Unless otherwise indicated, experiments were performed with 15-week-old GLP-1R KO mice [32] that were fed an autoclaved low-fat polysaccharide-rich chow diet (LabDiet 5021) *ad libitum*. KO mice were on C57BL/6J background and produced either from HetxHet or KOxKO breeding. KO mice were regularly backcrossed against C57BL/6J mice. WT littermates or C57BL/6J were used as controls. GF mice were maintained in flexible film isolators under a strict 12-hour light cycle and GF status was monitored regularly by anaerobic culturing and PCR for bacterial 16S rRNA. For Western-style diet experiment, 3 to 4-week-old or 10-week-old mice were fed an irradiated high-fat, high-sugar “Western” diet with 40% of calories from fat (Adjusted Fat Diet TD.96132, Envigo). All mouse experiments were performed using protocols approved by the Research Animal Ethics Committee in Gothenburg, Sweden.

### 2.2 Colonization of GF mice

For colonization with an unfractionated microbiota, the cecal content from an adult conventionally raised (CONV-R) C57BL/6J mouse was resuspended in 5 ml sterile PBS and 200 μl was administered by oral gavage to GF mice at weaning or 10 weeks-of-age. For each time-point, the colonization was performed in two batches with independent donors. The resulting conventionalized (CONV-D) mice were housed in individually ventilated cages under SPF-conditions.

### 2.3 Cecal water content

To calculate cecal water content glass tubes with cecal matter were weighed before and after an over-night lyophilization.

### 2.4 Histology and immunofluorescence

Proximal colon segments were fixed in 4% paraformaldehyde for at least 24 h, washed in PBS for 30 min and incubated in 70% Ethanol at 4 °C. The tissues were embedded in paraffin and sectioned to a thickness of 5 μm. To analyze histology, slides were stained with Hematoxylin and Eosin (H&E). For immunofluorescence staining we performed antigen retrieval by boiling slides in Citric acid buffer (10 mM, pH 6). For quantification of goblet cell number, we stained proximal colon tissue sections with Mucin-2 antibody and used Vectastain® ABC-AP and Vulcan Fast Red Chromogen Kit (Histolab) for detection. Sections were counterstained with Hematoxylin. Epithelial area was determined using ImageJ. For detection of apoptotic cells, we used the ApopTag® Plus In Situ Apoptosis Fluorescein Detection Kit (Merck) according to the manufacturer's instructions. For hypoxic staining we used the Hypoxyprobe™-Red549 kit (Hypoxyprobe) according to the manufacturer's instruction. Briefly we injected mice with 60 mg/kg body weight of Hypoxyprobe™ 1 h before harvest of tissues. Tissues were fixed in 4% paraformaldehyde for 24 h, washed in PBS 1 h and incubated in PBS-30% sucrose over-night before being embedded in OCT and frozen. Frozen sections were sectioned 10 μm thick and put on glass slides before staining with antibody against Hypoxyprobe™. Fluorescence images were captured using Zeiss Axioplan 2 microscope using the program Axio Vision 4.8.2.0 (Zeiss). Antibodies used in this study are listed in Supplementary Table 1.

### 2.5 Immunoblotting

Snap-frozen colon tissues were lysed in buffer A containing 50 mM Tris–HCl (pH 7.4), 150 mM NaCl, 1 mM EDTA, 1 mM Na_3_VO_4_, 20 mM NaF, 10 mM glycerophosphate, 1 mM PMSF, 10% glycerol, 1% Triton X-100 and EDTA-free protease inhibitor cocktail. For Western blotting, the lysates were sonicated and centrifuged at 20,000 g for 15 min at 4 °C, and the supernatant was mixed with 5× Laemmli buffer [0.156 M Tris–HCl (pH 6.8), 25% glycerol, 12.5% β-mercaptoethanol, 12.5% SDS, 0.1% bromophenol blue], followed by heating at 95 °C for 5–10 min. Samples treated with Laemmli buffer were separated in Bis-Tris gels (4–12% gradient), transferred to nitrocellulose membrane, blocked with 5% skim milk in TBS 0.05% Tween 20, and then probed with antibodies indicated at 4 °C overnight. The membrane was subsequently incubated with a secondary antibody labeled with HRP (horseradish peroxidase) enzyme and blots were detected by chemiluminescent substrates (Thermo Fisher). Blots were quantified with ImageJ. Antibodies used in this study are listed in Supplementary Table 1.

### 2.6 Transmission electron microscopy

The intestines were gently excised and flushed with PBS, followed by perfusion of modified Karnovsky's fixative (2.5% glutaraldehyde and 2% paraformaldehyde in 0.15 M Sodium cacodylate buffer) at a pressure equivalent to 10 cm of liquid for 5 min. Proximal colonic biopsies were selected, cut out and immersed in modified Karnovsky's for 24 h at 4 °C before being transferred to sodium cacodylate buffer. The specimens were post-fixed using osmium tetroxide for 2 h, washed and incubated for 1 h in 1% uranyl acetate followed by dehydrogenation in increasing ethanol concentrations: 70%-85%–95% and four times 5 min 99.5% ethanol. Specimens were transferred to acetone which was replaced three times prior to incubation in a 2:1 mixture of acetone and Agar 100 resin for 40 min. Samples were transferred to pure Agar 100 resin for 3–5 h at room temperature, placed in a mold and covered with fresh resin. Polymerization was performed at 40 °C for 15 h, followed by 60 °C for 48 h. The resulting blocks were sectioned for light microscopy. 60 nm thick sections were made using the Leica EM UC6 with a diamond knife (Diatom, Biel, Switzerland) and placed on hexagonal copper grids. Sections were visualized on a LEO912AB transmission electron microscope (Carl Zeiss). Images were acquired at 10,000× using an Olympus-SiS camera and stored using the iTEM software (Olympus-SiS, Münster, Germany). Images were subsequently analyzed in AxioVision Rel 4.6 with regards to mitochondrial shape. Mitochondrial shape was determined by calculating the circularity ratio [33] of 100 mitochondria per mouse. Briefly, the area (A) of the mitochondria was related to its perimeter (p) in the formula 4πA/p^2^.

### 2.7 Crypt isolation and seahorse

Freshly harvested proximal colon (2 cm) sections were collected on cold PBS and the luminal content was flushed twice with cold PBS. The attached fat and muscle layers were carefully removed to expose the intestinal epithelial layer. The intestine was opened longitudinally, and small incisions were made to facilitate digestion. The tissue was washed once with high glucose Dulbecco's modified eagle medium (DMEM) and further digested in a 0.03% collagenase solution in high glucose DMEM. The final digested suspension was centrifuged, the pellet was resuspended in high glucose DMEM with 10% fetal bovine serum (FBS) and filtered through a 70 μm nylon mesh. Crypts were pelleted and resuspended in a 10 μM Y27632 ROCK inhibitor solution in high glucose DMEM with 10% FBS. Crypts were seeded in a matrigel coated 96 well plate and cultured overnight.

The cultured crypts were analyzed with the Seahorse XF96 Analyzer to assess mitochondrial respiration and proton production through real-time extracellular acidification rate (ECAR) and oxygen consumption rate (OCR) analysis in combination with mitochondrial complex inhibitors (in order of injection, including 1.5 μM oligomycin to inhibit complex V (i.e., ATP synthase), 1 μM FCCP to uncouple the proton gradient, 0.5 μM antimycin A and rotenone to inhibit complex I and III). Three OCR and ECAR readings were analyzed after addition of each inhibitor and before automated injection of the subsequent inhibitor.

### 2.8 Colonic organoids

Colonic crypts were isolated by opening the proximal colon longitudinally, subsequently washing with PBS, followed by incubation with 5 mM EDTA containing PBS for 1 h. Around 200 crypts were seeded in a 20 μl matrigel drop and basal medium + WNT3A + EGF + Noggin + R-spondin (WENR) with 10 μM ROCK inhibitor (Sigma–Aldrich) was added. Medium was replaced every 2 days and passaging of organoids was done every 5–7 days. For WENR medium, 50 ng/ml EGF (Invitrogen), 100 ng/ml Noggin (Peprotech), 1 μg/ml R-spondin (R&D Systems) or R-spondin conditioned medium, B27 (Invitrogen), N2 (Invitrogen), 1.25 mM N-Acetylcysteine (Sigma–Aldrich) and 100 ng/ml WNT3A (R&D Systems) were dissolved in Advanced DMEM/F12 (Invitrogen) containing GlutaMAX, HEPES and Penicillin/Streptomycin.

### 2.9 Quantitative RT-PCR

Proximal colon tissues were homogenized in RLT buffer using a TissueLyzer (Qiagen). RNA was isolated using the RNeasy Kit with DNase I treatment (Qiagen). RNA was quantified using NanoDrop™ and cDNA was synthesized using the High-Capacity cDNA Reverse Transcription Kit (Applied Biosystems). qRT-PCR reactions were set-up in a 10 μl volume containing 1× SYBR Green Master Mix buffer (Thermo Scientific) and 900 nM gene-specific primers (or 500 nM L32 primers). Reactions were run on a CFX96 Real-Time System (Bio-Rad). Gene expression data were normalized to the ribosomal protein L32 using the ΔΔCT method. Oligonucleotides used in this study are listed in Supplementary Table 2.

### 2.10 Quantification of mtDNA copy number

Total DNA was isolated from colonic tissues using DNeasy Blood and Tissue Kit (Qiagen). The mtDNA content relative to nuclear DNA was assessed by qPCR. The reactions were set-up in a 25 μl volume containing 1× SYBR Green Master Mix buffer (Thermo Scientific) and 900 nM primers. Reactions were run on a CFX96 Real-Time System (Bio-Rad). mtDNA expression was normalized to the ribosomal protein single-copy nuclear gene using CO1 and Ndufv1 respectively as previously described [34] using the ΔΔCT method. Oligonucleotides used in this study are listed in Supplementary Table 2.

### 2.11 Statistical analysis

Data are presented as mean ± SEM. Each data point in the figures represent data from one mouse. Statistical differences were tested with Log-rank (Mantel–Cox) test, Student's *t* test, Mann–Whitney U test, one-way ANOVA or a two-way ANOVA with Tukey's or Dunnett's multiple comparison post hoc test as indicated in the figure legends. For comparison of two groups where n > 3, unpaired two-tailed Mann–Whitney U test was used and where n = 3 unpaired two-tailed unpaired Student's *t-*test was used. Statistical analysis was performed using GraphPad Prism 9.

## 3 Results

### 3.1 GF GLP-1R KO mice display a lethal phenotype with increased cecal water content

**Figure 1:**
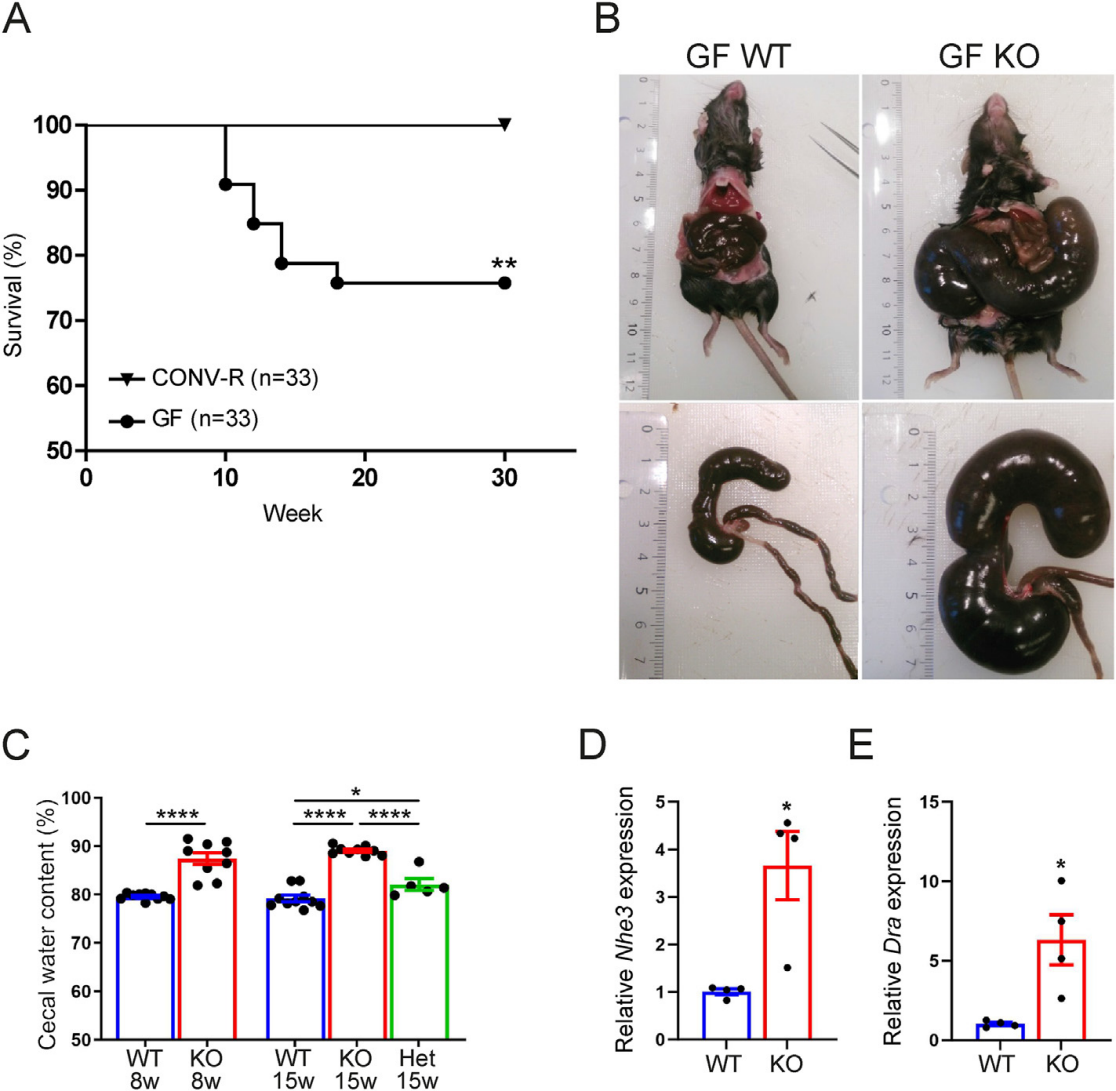
Lethal phenotype with increased cecal water content in GF GLP-1R KO mice. (A) Survival rate in GF and CONV-R KO mice (n = 33). (B) Gross morphology of GF WT and KO mouse abdomen. (C) Water content of cecal matter in 8w GF WT (n = 10) and KO (n = 9) mice and 15w WT (n = 10), KO (n = 9) and Het (n = 5) mice. (D–E) QPCR of *Nhe3* (D) and *Dra* (E) on proximal colon tissue from 15-week-old GF WT (n = 4) and KO (n = 4) mice. Data are presented as mean ± s.e.m. ∗p < 0.05, ∗∗p < 0.01, ∗∗∗∗p < 0.0001 determined by Log-rank (Mantel–Cox) test (A), Mann–Whitney U test (C: 8w, D, E) and one way ANOVA followed by Tukey's (C: 15w) multiple comparisons test. Except for A, all experiments were performed on male mice.

GLP-1 has important roles both locally and systemically in modulating host physiology and metabolism [35]. The gut microbiota regulate L-cell numbers and GLP-1 secretion both directly via metabolites signaling through GPCRs [23,26,28] and by providing energy substrates through SCFAs [29]. To further clarify the interaction between gut microbiota and GLP-1 signaling we rederived GLP-1R KO as GF (GF KO). Unexpectedly, 25% of the GF KO mice died between 10 and 18 weeks of age (Figure 1A), with no difference between male and female mice (Supplementary Figs. 1A and B). Mice that survived this critical time window survived until the end of the experimental observation period (30 weeks of age; Figure 1A and Supplementary Figs. 1A and B).

GF mice are known to have enlarged ceca compared with CONV-R counterparts [36]. Here we observed that in the absence of GLP-1R signaling the cecum was dramatically enlarged and constituted up to 50% of the body weight, even in mice that appeared healthy (Figure 1B). Furthermore, the cecal content was loose and contained more water compared to wild-type counterparts (Figure 1C). Interestingly, colonic expression of the genes encoding the apical ion transporters Na^+^/H^+^ exchanger 3 (NHE3) and the Cl^−^/HCO3^−^ exchanger Down-regulated in Adenoma (DRA), which are involved in ion and water re-uptake from the colon were upregulated in GF KO mice (Figure 1D,E).

### 3.2 GF GLP-1R KO mice display alterations in colonic morphology and loss of goblet cells

**Figure 2:**
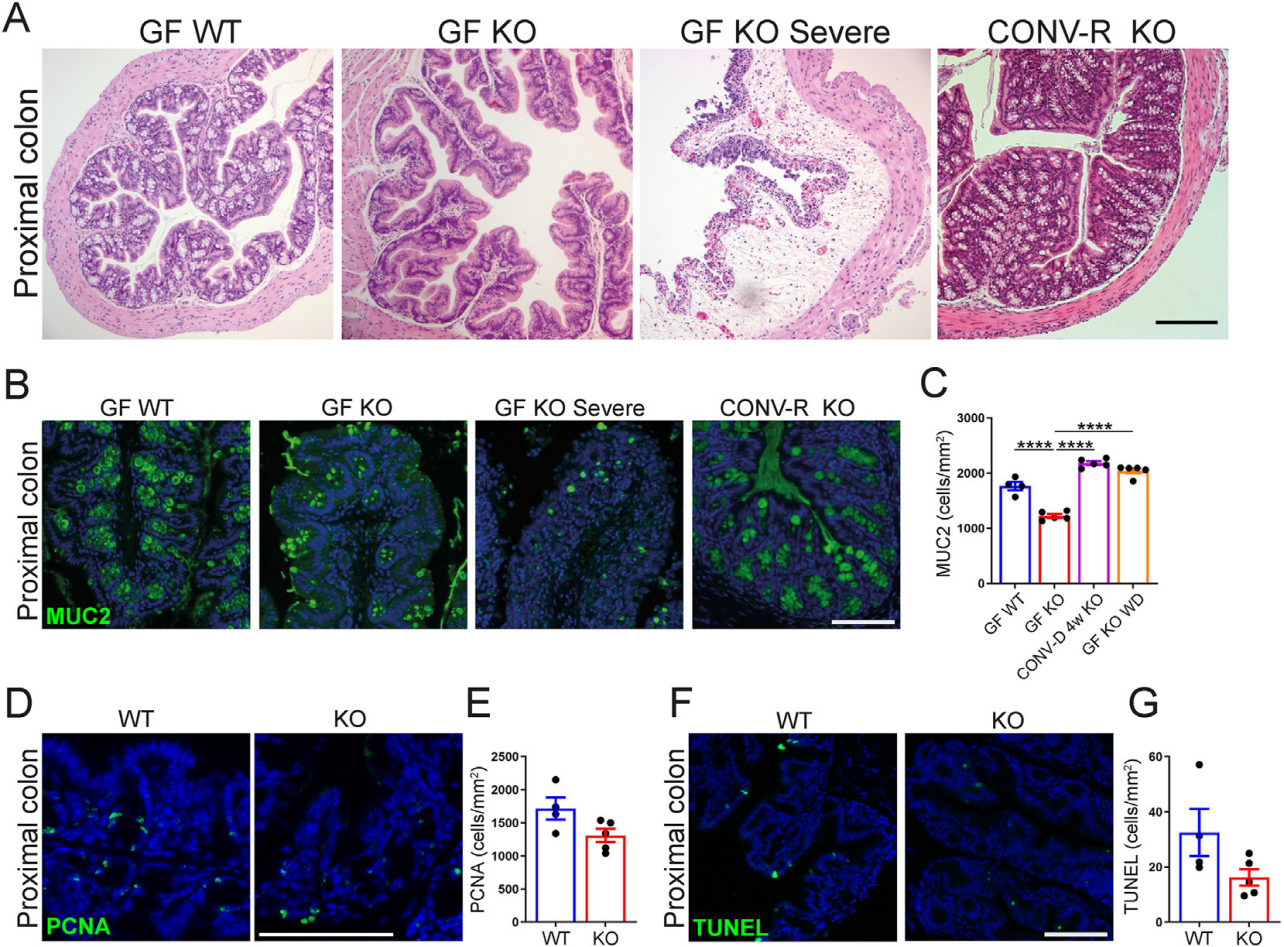
Colonic morphological alterations and loss of goblet cells in GF GLP-1R KO mice. (A) H&E staining of proximal colon sections from GF WT, GF KO, severely affected GF KO, and CONV-R KO mice. (B) Immunofluorescence staining of proximal colon sections from GF WT and KO, severely affected GF KO and CONV-R KO mice against MUC2 (green). (C) Quantification of MUC2 positive cells/epithelial tissue area in mice from 15-week-old GF WT (n = 4), GF KO (n = 5), KO colonized at weaning (CONV-D 4w KO) (n = 5) and GF KO fed a Western-style diet (GF KO WD) (n = 5). (D) Immunofluorescence staining of proximal colon sections from 15-week-old GF WT and KO, against PCNA (green). (E) Quantification of proliferating PCNA positive cells/epithelial tissue area in GF WT (n = 4) and KO (n = 5) mice. (F) TUNEL staining of proximal colon tissue from 15-week-old GF WT and KO mice. (G) Quantification of TUNEL positive cells/epithelial tissue area in GF WT (n = 4) and KO (n = 5) mice. Data are presented as mean ± s.e.m. ∗∗∗∗p < 0.0001 determined by one way ANOVA followed by Dunnett's (C, comparing against GF KO) multiple comparisons test. Nuclei were stained with Hoechst 33342 (blue) (B,D,F). Scale bar represents 200 μm (A) and 100 μm (B,D,F). All experiments were performed on male mice.

Next, we compared the morphology of proximal colon in GF KO mice with severe disease euthanized before they were about to die naturally (GF KO Severe) and 15-week-old grossly normal GF KO mice. GF KO Severe mice exhibited infiltration of red blood cells into the tissue, loss of MUC2+ goblet cells, and distortion of epithelial structure (Figure 2A,B). In contrast, the grossly normal GF KO mice had intact epithelium but fewer goblet cells in the proximal colon (Figure 2A–C and Supplementary Fig. 1C) whereas colonic endocrine cell numbers were not different between GF KO vs. WT mice (Supplementary Fig. 1D,E). To investigate if the changes in the colonic epithelium reflected differences in cell turnover, we investigated markers of proliferation and apoptosis in GF KO mice. However, we did not observe any differences in the number of PCNA + proliferating cells or changes in expression of the gene encoding the proliferation marker Ki67 (Figure 2D,E and Supplementary Fig. 1F) or number of apoptotic cells (Figure 2F,G). Heterozygous mice had slightly increased water content in the cecum (Figure 1C) but showed no other morphological alterations in proximal colon (Supplementary Fig. 1G).

### 3.3 Colonization with complete microbiota or high caloric diet feeding prevents colonic phenotypes in GF GLP-1R KO mice

**Figure 3:**
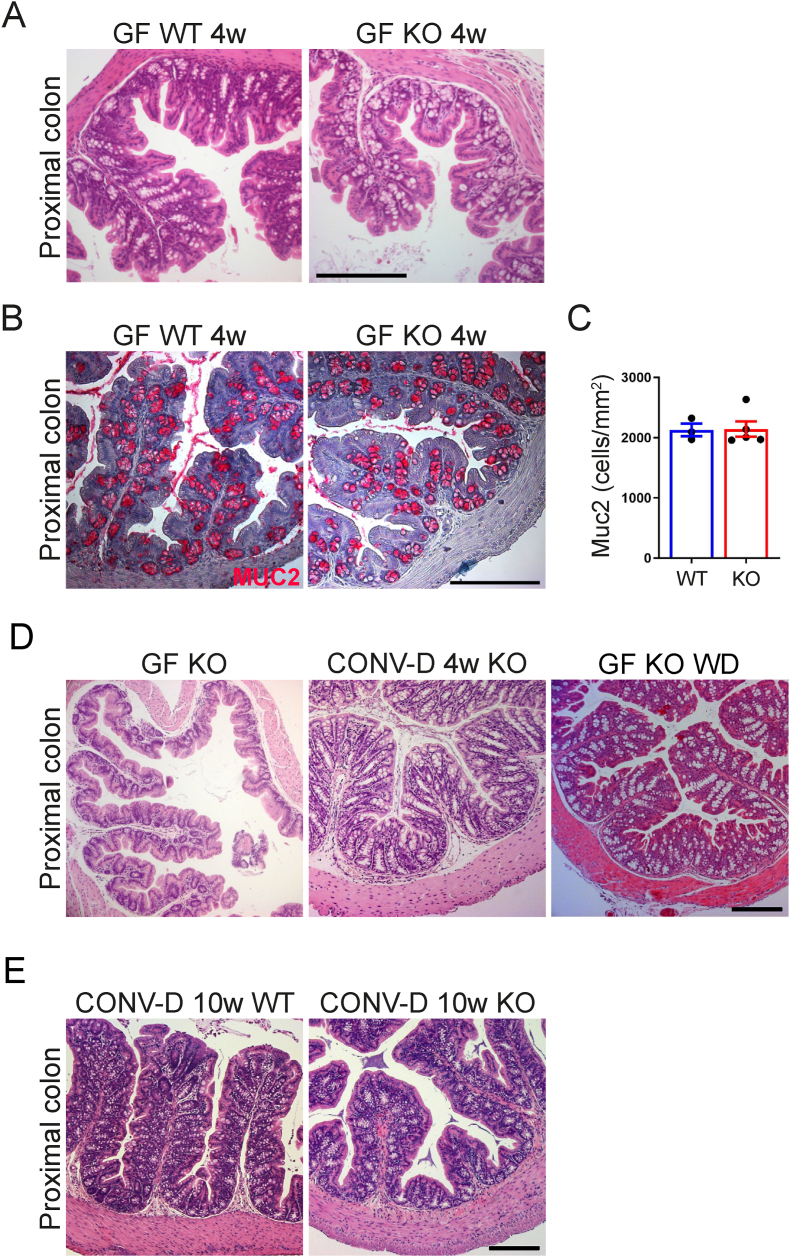
Colonization or high caloric diet prevents phenotypes in GF GLP-1R KO mice. (A) H&E staining of proximal colon sections from GF WT and GF KO mice at 4 weeks of age. (B) Staining of proximal colon sections from GF WT and KO mice 4 weeks of age against MUC2 (red) and Hematoxylin (blue). (C) Quantification of MUC2 positive cells/epithelial tissue area in male and female GF WT (n = 4) and KO (n = 5) mice 4 weeks of age. (D) H&E staining of proximal colon sections from 15-week-old male KO mice that are either GF, conventionalized at weaning (CONV-D 4w) or fed a Western-style diet (GF KO WD) from weaning. (E) H&E staining of proximal colon sections from 30-week-old WT or KO male mice that were conventionalized at 10 weeks (CONV-D 10w). Data are presented as mean ± s.e.m. Scale bar represents 200 μm.

To further investigate how early colonic phenotypes were observed, we first analyzed mice immediately after weaning at 4 weeks of age. At this time-point we did not observe any differences in morphology or goblet cell numbers (Figure 3A–C). Similarly, the epithelium had normal intestinal morphology at 8 weeks of age (not shown), although these mice had increased cecal water content (Figure 1C).

To investigate if a complete microbiota could prevent the phenotypes, we colonized GF KO mice with a normal mouse microbiota from an adult CONV-R C57BL/6J mouse at different time-points. KO mice colonized at weaning (CONV-D 4w KO) had normal intestinal morphology when analyzed at 15 weeks of age (Figure 3D) and goblet cell numbers were increased compared with GF KO mice (Figure 2C and Supplementary Fig. 1C). Similarly, colonization at 10 weeks of age, just prior to the first deaths, completely prevented the lethal phenotype observed in GF KO mice (n = 14/14 surviving mice) and CONV-D KO mice had similarly normal colonic morphology as exhibited by WT mice at the end of the experiment (Figure 3E).

We have previously demonstrated that normalization of the energy availability in GF mice by either supplementation of butyrate, dietary fat or microbial colonization normalizes GLP-1 production and intestinal transit [29]. Thus, we next fed GF KO mice an irradiated Western-style diet rich in fat and sucrose (GF KO WD) to investigate if increased energy availability in the absence of a microbiota also prevented lethality and morphological changes in the colonic epithelium in GF KO mice. We observed that feeding KO mice a Western-style diet from 10 weeks of age completely reversed the lethal phenotype previously exhibited by conventional diet fed GF KO mice in two independent experiments (terminated after 23 weeks (n = 7/7 survived) and after 30 weeks (n = 8/8 survived), respectively). Both colonic morphology (Figure 3D) and goblet cell number (Figure 2C and Supplementary Fig. 1C) were normalized in GF KO mice fed Western-style diet from weaning to 15 weeks of age. Thus, our data suggests that GLP-1R signaling is indispensable for maintaining gut homeostasis in germ-free mice, which are energy deprived, and that lethality can be rescued by reintroducing a gut microbiota or feeding the mice with a Western-style diet.

### 3.4 GF GLP-1R KO mice display mitochondrial fragmentation and increased oxygen levels in the colon

**Figure 4:**
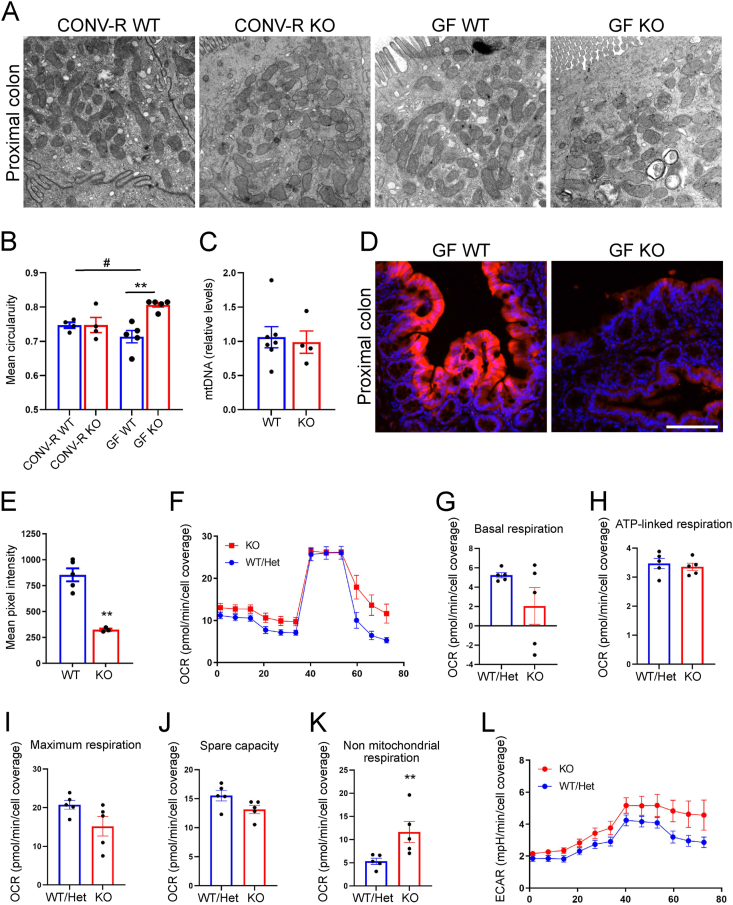
GF GLP-1R KO mice are associated with mitochondrial fragmentation and increased oxygen levels in the colon. (A) TEM images of proximal colon from 15-week-old CONV-R WT, CONV-R KO, GF WT and GF KO mice. (B) Quantification of circularity of mitochondria in colonocytes of 15-week-old CONV-R WT and KO (n = 4) and GF WT and KO (n = 5) mice. (C) Quantification of mitochondrial DNA in tissue from proximal colon of GF WT (n = 7) and KO (n = 4) mice. (D) Proximal colon tissue from GF WT and KO mice stained against hypoxyprobe. (E) Quantification of mean pixel intensity of hypoxyprobe in tip of epithelium in GF WT (n = 5) and KO (n = 3) mice. (F) OCR values during a Seahorse mitochondrial stress test (n = 5). Quantification of Basal respiration (G), ATP-linked respiration (H), Maximum respiration (I), Spare capacity (J) and Non-mitochondrial respiration (K). (L) ECAR values during a Seahorse mitochondrial stress test (n = 5). Data are presented as mean ± s.e.m. ∗p < 0.05, ∗∗p < 0.01, #Significant for genotype–colonization interaction determined by two-way ANOVA followed by Tukey's multiple comparisons test (B), Student's *t* test (E) and Mann–Whitney U test (K). Nuclei were stained with Hoechst 33342 (blue) (D). Scale bar represents 100 μm. All experiments were performed on male mice.

The observation that increasing energy levels in colon prevented the lethality and morphological changes in GF KO mice, prompted us to investigate mitochondrial morphology and function in the colon. Transmission electron microscopy showed fragmentation of mitochondria in GF KO mice compared with GF WT mice (Figure 4A), associated with increased circularity of mitochondria in GF KO mice compared with WT counterparts, which was not observed in conventionally raised KO mice (Figure 4B). The altered mitochondrial morphology was not associated with any changes in mitochondrial DNA levels (Figure 4C).

An excessive fragmentation of mitochondria can be associated with impaired mitochondrial function [37,38] and reduced respiration leading to increased oxygen levels. Consistent with these findings, the colonic epithelium was more oxygenated (e.g. less hypoxic) in GF KO compared with GF WT colon (Figure 4D,E). Taken together our data suggest that GF KO mice have impaired mitochondrial morphology and function.

To further analyze cellular metabolism and mitochondrial function in GF KO colon we performed a Seahorse mitochondrial stress test in isolated colonic crypts. We did not observe any major changes in oxygen consumption rate (OCR) (Figure 4F–J) comparing KO with WT and Het crypts. However, non-mitochondrial respiration, measured by oxygen consumption when the mitochondrial electron transport chain is fully inhibited, was increased in GF KO colonic crypts (Figure 4K) [39]. Furthermore, we observed a trend (p = 0.13) towards higher extracellular acidification rate (ECAR) in the KO group, consistent with increased glycolytic activity in GF KO mice (Figure 4L). In summary, the data suggests that loss of GLP-1R signaling is associated with dysfunctional mitochondrial adaptation under GF conditions.

### 3.5 Loss of adaptation to energy deprivation in GF GLP-1R KO mice

**Figure 5:**
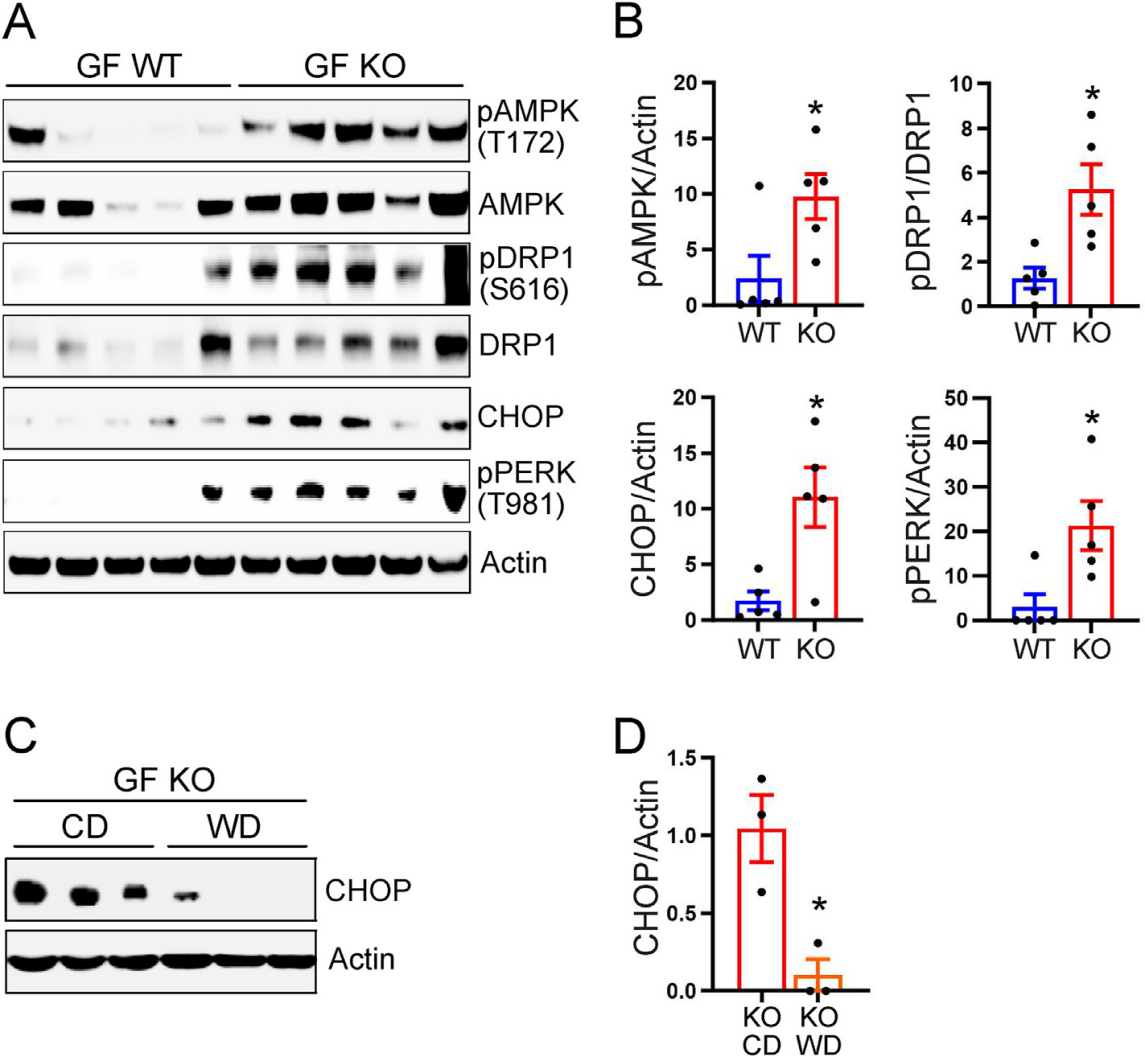
Loss of adaptation to energy deprivation in GF GLP-1R KO mice. (A) Total protein extracts from proximal colon analyzed by immunoblotting with antibodies against pAMPK, AMPK, pDRP1, DRP1, CHOP, pPERK and Actin. (B) Relative quantification of proteins analyzed in (A) normalized to Actin (n = 5). (C) Total protein extracts from proximal colon of mice fed chow diet (CD) or Western-style diet (WD) were analyzed by immunoblotting with antibodies against CHOP and Actin. (D) Relative quantification of proteins analyzed in (C) normalized to Actin (n = 3). Data are presented as mean ± s.e.m. ∗p < 0.05, determined by Mann–Whitney U test (B) or Student's *t* test (D). All experiments were performed on male mice.

To provide potential insights in molecular mechanisms underlying the energy-deprived state we first investigated the levels of AMP-activated protein kinase (AMPK) phosphorylation at Thr 172, which has been previously associated with energy deprivation [40,41]. Consistent with the hypothesis that GF KO mice suffered from energy deprivation we observed increased levels of Thr172 phosphorylation of AMPK in the colon of GF KO compared with GF WT (Figure 5A,B). Interestingly, AMPK can mediate mitochondrial fission in response to energy stress [42], which is supported by increased levels of Dynamin-related protein 1 (DRP1) phosphorylation at Ser616 (Figure 5A,B). This finding is consistent with the increased mitochondrial fragmentation in GF KO mice (Figure 4A,B).

Energy deprivation as well as mitochondrial dynamics are both associated with ER-stress [[43], [44], [45], [46]] and accordingly we observed higher levels of both CCAAT/enhancer-binding protein homologous protein (CHOP) and phosphorylated protein kinase RNA-like endoplasmic reticulum kinase (pPERK) in GF KO mice, suggesting unresolved ER stress (Figure 5A,B). To investigate if these phenotypes could be associated with energy deprivation we next investigated if ER stress is reduced in GF KO mice fed a Western-style diet. The levels of CHOP decreased in the colon of GF KO mice on Western-style diet feeding (Figure 5C,D), which suggests that during GF conditions GLP-1R signaling is important for preventing energy deprivation and associated stress responses in the colon.

### 3.6 Loss of stemness after differentiation in GF GLP-1R KO colon

**Figure 6:**
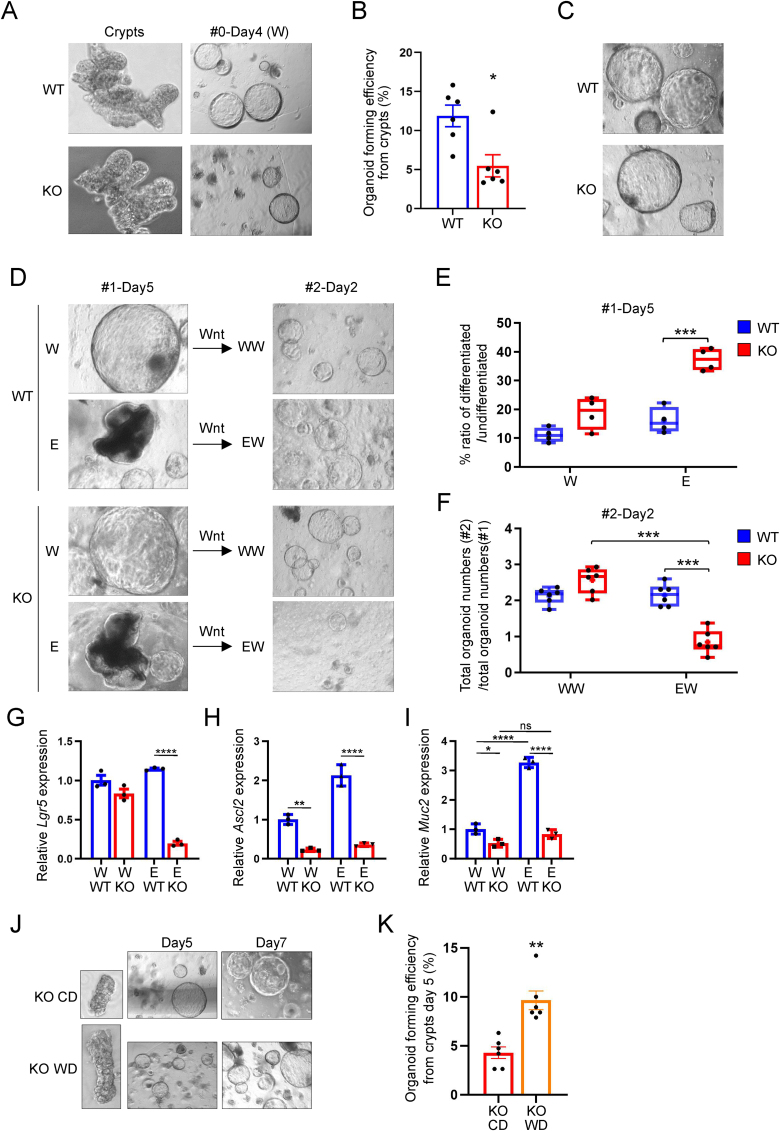
Loss of stemness after differentiation in GF GLP-1R KO colon. (A) Isolated crypts and cultured organoids 4 days after isolation in WENR. (B) Organoid forming efficiency of colonic crypts from GF WT and KO mice in the maintenance medium (WENR, W) (n = 6 mice). (C) Organoids 4 days after passaging of organoids in A in WENR. (D) Organoids in C were cultured in the presence (W) or absence (differentiation medium, E) of Wnt3A (#1-Day5), which were reintroduced with Wnt3A after passaging (#2-Day2). (E) % ratio of differentiated/undifferentiated organoids in D, #1-Day5 (n = 4). (F) Propagating ability of organoids were assessed by counting total organoid numbers in the 7th day of passage 1 and those in the 2nd day of passage 2 (n = 6). QPCR of *Lgr5* (G), *Ascl2* (H) and *Muc2* (I) from organoids grown in presence (WENR) or absence (ENR) of Wnt (n = 3 per condition and genotype). (J) Isolated crypts and cultured organoids from GF KO mice fed either chow diet (CD) or Western-style diet (WD). (K) Organoid forming efficiency of colonic crypts from GF KO mice fed either CD or WD in the maintenance medium (WENR, W) (6 wells from 2 mice/condition). Data are presented as mean ± s.e.m. ∗p < 0.05, ∗∗p < 0.01, ∗∗∗p < 0.001, ∗∗∗∗p < 0.0001, determined by Mann–Whitney U test (B, K) Student's *t* test (G, H) and two-way ANOVA followed by Tukey's multiple comparisons test (E, F, I). All experiments were performed on tissues from male mice.

Changes in mitochondrial function and ER-stress can result in loss of stemness [47,48] and thus we generated organoids from the colonic crypts isolated from GF WT or KO mice to investigate if GLP-1R deficiency affects colonic stem cell properties (Figure 6A). Interestingly, GF KO colonic crypts had reduced organoid forming efficiency compared with crypts from GF WT mice (Figure 6B). However, surviving organoids from the crypts from GF KO colon were successfully passaged (Figure 6C), allowing us to investigate if the differentiation potential varied between genotypes. ER stress is associated with rapid loss of intestinal stem cells via accelerated differentiation from stem cells to transit amplifying cells [48]. As intestinal organoids with fewer budding structures (cystic organoids) tend to exhibit less differentiation and higher proliferation rates [[48], [49], [50]], we assessed the ratio between less cystic (more differentiated) and cystic (undifferentiated) organoids to evaluate the impact on intestinal stemness upon removal of the stem cell maintenance factor Wnt family member 3A (Wnt3A) from the media (differentiation condition, designated as E).

Upon Wnt3A removal, GF KO organoids displayed a reduced production of cystic organoids (indicating increased differentiation) compared to GF WT organoids (Figure 6D,E). Further investigation tested if differentiated KO organoids retained their ability to propagate after passaging in maintenance media. Organoids cultured in the presence of Wnt3A successfully underwent passaging irrespective of their genotypes (WW, Figure 6D,F). However, differentiated GF KO organoids failed to propagate after passaging, even upon reintroduction of Wnt3A (EW) (Figure 6D,F). These findings suggest that GLP-1R deficiency resulted in a loss of stemness, akin to previous research demonstrating the loss of stem cells due to differentiation under ER stress [48]. This conclusion is supported by the reduced expression of genes encoding the stem cell markers Leucine-rich repeat-containing G-protein coupled receptor 5 (*Lgr5*) and Achaete scute-like 2 (*Ascl2*) observed after Wnt3a removal, and the decreased expression of *Ascl2* even in the presence of Wnt3a (Figure 6G,H). To investigate whether the phenotype of reduced goblet cell number observed *in vivo* could be explained by altered differentiation we analyzed the expression of the goblet cell characteristic mRNA, *Muc2*. Indeed, we observed lower *Muc2* expression in organoids from GF KO both in presence and absence of Wnt and the increase in *Muc2* expression after removal of Wnt that we observed in GF WT organoids was completely abolished in organoids from GF KO mice (Figure 6I).

Finally, we wanted to investigate if increasing energy availability through Western-style diet feeding would also affect stem cell properties. Indeed, organoid forming efficiency was elevated in the crypts from GF KO fed Western-style diet, compared to those fed chow diet (Figure 6J,K), supporting the observations that Western-style diet prevents both lethality, colonic ER-stress and goblet cell loss in GF KO mice.

## 4 Discussion

GLP-1 is primarily known for its role as an incretin hormone although some studies have identified more local effects in the gut [11,12]. The gut microbiota and their resulting metabolites can modulate GLP-1 production through activation of GPCRs [15]. However, microbial production of SCFAs is an important source of energy for the colonic mucosa and we previously demonstrated that GF mice have increased basal levels of GLP-1, which reduces small intestinal transit to potentially allow increased time for energy extraction [29]. Thus, in contrast to the well-established pharmacological effects of appetite regulation which are observed under conditions of energy excess, the paracrine effects of GLP-1 to enhance energy extraction is a physiological response to energy deficiency in the colon. Moreover, *Glp1r*^−/−^ mice exhibit impaired mucosal defence, and enhanced sensitivity to gut epithelial injury [12]. To extend these findings we rederived GLP-1R KO as GF. Surprisingly, 25% of the mice died before 18 weeks of age, a phenotype associated with a grossly enlarged cecum, increased cecal water content, loss of mucosal structure and loss of goblet cells.

The effects of loss of GLP-1R signaling in the gastrointestinal tract including increased water content are consistent with that the most frequent adverse events following GLP-1R agonist treatments are gastrointestinal disorders including constipation [2,6]. However, the molecular mechanisms for increased water content in the distal gut in the absence of GLP-1R signaling is at present unknown. The increased water content in the colon requires increased water and ion re-absorption, which is consistent with increased expression of the apical ion transporters NHE3 and DRA. These ion channels are dependent on an active basolateral Na^+^K^+^-ATPase that is pumping absorbed Na^+^ ions out of the cell. Interestingly, the water and ion transport through Na^+^K^+^-ATPase is energy demanding and during the limited energy availability under GF conditions it may contribute to the severity of energy deprivation.

Based on our findings, we hypothesize that the loss of goblet cells in the proximal colon may contribute to the loss of intestinal integrity since reduced mucus production may increase shear stress and thus damage of the epithelium. This is consistent with observations that mice deficient in Mucin 2 (MUC2), the main component of colonic mucus in humans and mice, develop spontaneous colitis and premature death upon infectious colitis [51,52]. Importantly, not all GF KO mice died although all GF KO mice analyzed had reduced goblet cell numbers at 15 weeks, which suggests that there may be a threshold of goblet cell number below which the severe phenotypes develop.

Interestingly, GF KO mice develop normally before weaning and the lack of phenotype early in life may be explained by the fact that the pups are fed energy rich milk from their mothers which supplies sufficient energy to the colonic mucosa to prevent alterations. This further suggests that energy deprivation, such as under GF conditions, initiates the gastrointestinal phenotypes. Interestingly, colonic pathology could be prevented by colonization with a normal mouse microbiota or by feeding the mice with energy-rich Western-style diet before (4 weeks of age) or after phenotypic changes occur (10 weeks of age).

Consistent with energy deprivation, GF mice have increased levels and activity of the metabolic sensor AMPK [41]. Here we show that AMPK activation is even more strongly induced in the absence of GLP-1R signaling in GF KO mice indicating that the colonic epithelium is markedly energy-deficient. This could potentially be attributed to deficiency in oxidative metabolism. In agreement with calorie restriction [40,53], which has been shown to induce ER-stress [[43], [44], [45]], we observed that GF KO mice had increased levels of the ER-stress markers CHOP and pPERK in the colon compared to WT counterparts. These phenotypes were reversed upon feeding mice with Western style diet, which further suggests that the ER stress observed in KO mice is caused by reduced energy availability. Taken together, our data thus suggest that during energy-deprived situations, such as under germ-free conditions, GLP-1R signaling maintains energetically costly cellular processes such as mucus production to maintain intestinal integrity.

Increased ER-stress has been shown to affect stem cells by reducing stemness [48]. In agreement, the organoid forming efficiency is reduced in GF KO colon suggesting that stem cell function is impaired. Organoids from GF KO mice are less stable in their undifferentiated state, since we observed increased differentiation in GF KO organoids when Wnt3A is removed. An altered differentiation may also affect cell fate decisions and we observed reduced *Muc2* expression in GF KO organoids, suggesting that lack of GLP-1R signaling cause goblet cell differentiation to become dysregulated. Taken together our findings suggest that the energy deprivation in the GF KO colon has consequences on stem cell properties that remain evident *ex vivo* and accordingly, feeding GF KO mice Western-style diet for one week increased the organoid forming efficiency. In agreement with our findings, GLP-1R signaling has been shown to affect stem cells in the gut [11], and GLP-1R signaling may promote repair mechanisms in the colon [12] that further may contribute to restoring and maintaining colonic integrity.

In summary, we show that GLP-1R signaling is important for maintaining colonic integrity during conditions where the availability of energy is limited. In the absence of GLP-1R there is increased water content and reduced mitochondrial respiration, as illustrated by increased oxygen levels in the energy-deprived colonic mucosa. Dysregulated metabolism can contribute to increased ER-stress and altered stem cells properties that may lead to reduced goblet cell differentiation further enhancing the colonic phenotypes. Although GF mice are a model system, restoring nutrition in children with intestinal failure through parenteral nutrition reduces circulating GLP-1 levels [54], suggesting that the L cell functions as a local energy sensor, linking GLP-1 secretion to the state of gastrointestinal energy availability. It remains to be investigated if GLP-1 signaling can contribute to protection against developing of environmental enteropathy; which is characterized by alterations in intestinal structure, function, and cause immune activation, and can contribute to childhood undernutrition [55].

Taken together, we identified new roles for GLP-1R signaling locally in the colon. Considering the increasing interest in the actions of GLP-1-based therapies, extending our knowledge about the function of GLP-1 in the gut may help to identify new mechanisms linking GLP-1R signaling to the control of mucosal immunity, and the integrity and function of the gut mucosal epithelium.

## Funding

This study was supported by the Novo Nordisk Foundation (NNF15OC0016798 and NNF17CO0028232), Knut and Alice Wallenberg Foundation (2017.0026), the Swedish Research Council (2013–07800), the Leducq Foundation (17CVD01), EFSD and Novo Nordisk A/S Programme for Diabetes Research in Europe (94867), and grants from the Swedish state under the agreement between the Swedish government and the county councils, the ALF-agreement (ALFGBG-718101). F.B. is the Torsten Söderberg Professor in Medicine and a Wallenberg Scholar. A.K. is supported by the National Research Foundation of Korea (NRF) grant funded by the Korea government (MSIT) (No. 2023R1A2C1002876) and by the Korean Fund for Regenerative Medicine (KFRM) grant funded by the Korea government (the Ministry of Science and ICT, the Ministry of Health & Welfare) (KFRM 22A0301L1). D.J.D. is supported by CIHR Foundation grant 154321, a Banting and Best Diabetes Centre-Novo Nordisk Chair in Incretin Biology, and the Sinai Health Novo Nordisk Fund in regulatory peptides.

## CRediT authorship contribution statement

**Thomas U. Greiner:** Writing – original draft, Visualization, Methodology, Investigation, Formal analysis, Conceptualization. **Ara Koh:** Writing – review & editing, Methodology, Investigation. **Eduard Peris:** Writing – review & editing, Methodology, Investigation. **Mattias Bergentall:** Writing – review & editing, Methodology, Investigation. **Malin E.V. Johansson:** Writing – review & editing, Methodology. **Gunnar C. Hansson:** Writing – review & editing, Supervision, Investigation. **Daniel J. Drucker:** Writing – review & editing, Methodology. **Fredrik Bäckhed:** Writing – original draft, Supervision, Funding acquisition, Conceptualization.

## Declaration of competing interest

F.B. receives research support from Biogaia AB, is founder and shareholder of Implexion Pharma AB, Roxbiosens Inc, and on the scientific advisory board for Bactolife A/S. D.J.D. has served as a consultant or speaker within the past 12 months to Altimmune, Amgen, Kallyope, Merck Research Laboratories, Novo Nordisk Inc., Pfizer Inc. and Sanofi Inc.. Neither D.J.D. nor his family members hold issued stock directly or indirectly in any of these companies. D.J.D holds non-exercised options in Kallyope.

## Data Availability

Data will be made available on request.

## Appendix A Supplementary data

Figure S1 Survival rate in GF and CONV-R KO female (A) and male (B) mice from Figure 1. (C) Staining of proximal colon sections from GF WT, GF KO, CONV-D 4w KO and GF KO WD mice against MUC2 (red) and Hematoxylin (blue). (D) Immunofluorescence staining of proximal colon sections from GF WT and KO mice against ChrgA (red). (E) Quantification of ChrgA positive cells/epithelial tissue area in mice from 15-week-old GF WT (n = 4) and GF KO (n = 5). (F) QPCR of *Mki67* on proximal colon tissue from 15-week-old male GF WT and GF KO mice. (G) H&E staining of proximal colon from GF GLP-1R Het male mouse. Data are presented as mean ± s.e.m. Nuclei were stained with Hoechst 33342 (blue) (D). Scale bars represent 100 μm (D) and 200 μm (C,G). Figure S1
