# Supplementary figures and images for: GLP-1R signaling modulates colonic energy metabolism, goblet cell number and survival in the absence of gut microbiota
Source: Mol Metab. 2024 Mar 21;83:101924. doi: 10.1016/j.molmet.2024.101924 (PMC11002751; doi:10.1016/j.molmet.2024.101924)

# Supplementary Figure 1

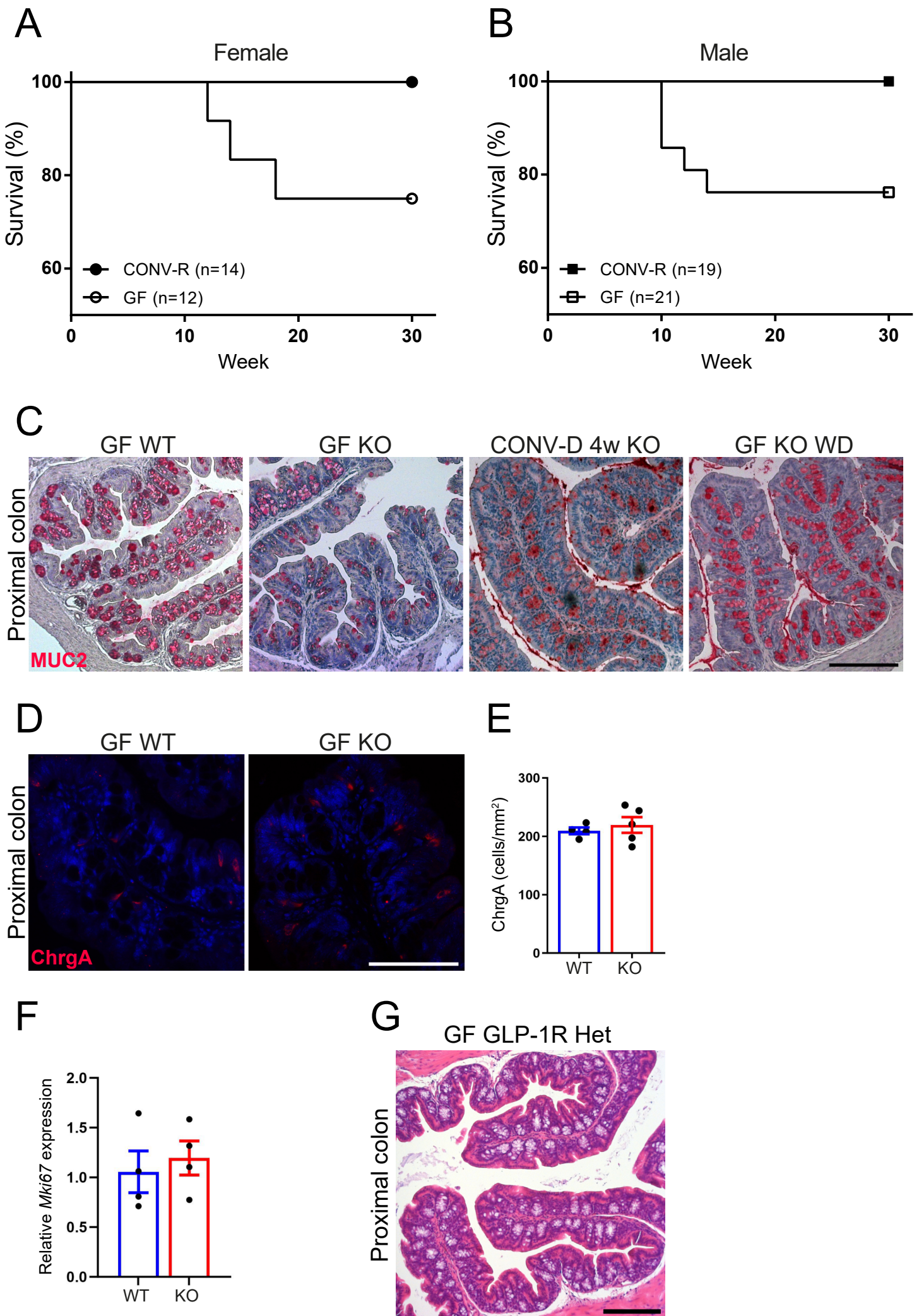

Supplement: Figure S1 — Survival rate in GF and CONV-R KO female (A) and male (B) mice from Figure 1. (C) Staining of proximal colon sections from GF WT, GF KO, CONV-D 4w KO and GF KO WD mice against MUC2 (red) and Hematoxylin (blue). (D) Immunofluorescence staining of proximal colon sections from GF WT and KO mice against ChrgA (red). (E) Quantification of ChrgA positive cells/epithelial tissue area in mice from 15-week-old GF WT (n = 4) and GF KO (n = 5). (F) QPCR of Mki67 on proximal colon tissue from 15-week-old male GF WT and GF KO mice. (G) H&E staining of proximal colon from GF GLP-1R Het male mouse. Data are presented as mean ± s.e.m. Nuclei were stained with Hoechst 33342 (blue) (D). Scale bars represent 100 μm (D) and 200 μm (C,G). [file mmc1.pdf]
