# Supplementary material for: GLP-1R signaling modulates colonic energy metabolism, goblet cell number and survival in the absence of gut microbiota
Source: Mol Metab. 2024 Mar 21;83:101924. doi: 10.1016/j.molmet.2024.101924 (PMC11002751; doi:10.1016/j.molmet.2024.101924)
Supplement: Multimedia component 2 [file mmc2.docx]

**Supplementary table 1**

| **Antibodies** | **SOURCE** | **IDENTIFIER** |
| --- | --- | --- |
| AlexaFluor donkey anti-rabbit IgG 488 | Thermo Fisher Scientific | RRID: AB_2535792 Cat# a-21206 |
| AlexaFluor donkey anti-rabbit IgG 594 | Thermo Fisher Scientific | RRID: AB_141637 Cat# a-21207 |
| Hoechst 33342 | Thermo Fisher Scientific | Cat# H1399 |
| Mucin-2 | Hansson Gunnar [1] | N/A |
| Chromogranin A | Immunostar | Cat# 20085 |
| Goat Anti-Rabbit IgG Antibody (H+L), Biotinylated | Vector Laboratories | BA-1000-1.5 |
| PCNA | Santa Cruz | Cat# sc-7907 |
| AMPK | Cell Signaling Technology | Cat# 2532 |
| pAMPK | Cell Signaling Technology | Cat# 2535 |
| CHOP | Cell Signaling Technology | Cat# 2895 |
| pPERK | Santa Cruz | Cat# sc-32577 |
| Drp1 | BD Biosciences | Cat# 611738 |
| pDrp1 | Cell Signaling Technology | Cat# 3455 |
| Actin | Cell Signaling Technology | Cat# 4970 |

[1] Johansson, M.E., Phillipson, M., Petersson, J., Velcich, A., Holm, L., Hansson, G.C., 2008. The inner of the two Muc2 mucin-dependent mucus layers in colon is devoid of bacteria. Proc Natl Acad Sci U S A 105(39):15064-15069.
