# Supplementary material for: GLP-1R signaling modulates colonic energy metabolism, goblet cell number and survival in the absence of gut microbiota
Source: Mol Metab. 2024 Mar 21;83:101924. doi: 10.1016/j.molmet.2024.101924 (PMC11002751; doi:10.1016/j.molmet.2024.101924)
Supplement: Multimedia component 3 [file mmc3.docx]

**Supplementary table 2**

| **Oligonucleotides** | **Source** |
| --- | --- |
| Ascl2F CGCTGCCCAGACTCATGCCC | [1] |
| Ascl2R GCTTTACGCGGTTGCGCTCG | [1] |
| Lgr5F ACCCGCCAGTCTCCTACATC | [2] |
| Lgr5R GCATCTAGGCGCAGGGATTG | [2] |
| Muc2F CCCAGAAGGGACTGTGTATG | [3] |
| Muc2R TTGTGTTCGCTCTTGGTCAG | [3] |
| **Nhe3F GCACAGAAGCGGAGGAATAG** |  |
| **Nhe3R TCATAGTTGGTGGCCTCCTC** |  |
| **Dra F TTGCTCTGCTGGTCAACATC** |  |
| **Dra R TGTAACGACAACTCCCACCA** |  |
| **Mki67F CAATGTGCCTCGCAGTAAGA** | [4] |
| **Mki67R GCATCTTTGGGGTTTTCTCA** | [4] |
| L32F CCTCTGGTGAAGCCCAAGATC | [5] |
| L32R TCTGGGTTTCCGCCAGTTT | [5] |
| CO1F TGCTAGCCGCAGGCATTA | [6] |
| CO1R GGGTGCCCAAAGAATCAGAAC | [6] |
| Ndufv1F CTTCCCCACTGGCCTCAAG | [6] |
| Ndufv1R CCAAAACCCAGTGATCCAGC | [6] |

[1] Yang, J., Lin, X., Pan, Y., Wang, J., Chen, P., Huang, H., et al., 2016. Critical roles of mTOR Complex 1 and 2 for T follicular helper cell differentiation and germinal center responses. Elife 5.

[2] Basak, O., Beumer, J., Wiebrands, K., Seno, H., van Oudenaarden, A., Clevers, H., 2017. Induced Quiescence of Lgr5+ Stem Cells in Intestinal Organoids Enables Differentiation of Hormone-Producing Enteroendocrine Cells. Cell Stem Cell 20(2):177-190 e174.

[3] Bergstrom, A., Kristensen, M.B., Bahl, M.I., Metzdorff, S.B., Fink, L.N., Frokiaer, H., Licht, T.R., 2012. Nature of bacterial colonization influences transcription of mucin genes in mice during the first week of life. BMC Res Notes 5:402.

[4] Sommer, F., Backhed, F., 2015. The gut microbiota engages different signaling pathways to induce Duox2 expression in the ileum and colon epithelium. Mucosal Immunol 8(2):372-379.

[5] Molinaro, A., Caesar, R., Holm, L.M., Tremaroli, V., Cani, P.D., Backhed, F., 2017. Host-microbiota interaction induces bi-phasic inflammation and glucose intolerance in mice. Mol Metab 6(11):1371-1380.

[6] Haemmerle, G., Moustafa, T., Woelkart, G., Buttner, S., Schmidt, A., van de Weijer, T., et al., 2011. ATGL-mediated fat catabolism regulates cardiac mitochondrial function via PPAR-alpha and PGC-1. Nat Med 17(9):1076-1085.
